# Supplementary material for: Global hypo-methylation in a proportion of glioblastoma enriched for an astrocytic signature is associated with increased invasion and altered immune landscape
Source: eLife. 2022 Nov 22;11:e77335. doi: 10.7554/eLife.77335 (PMC9681209; doi:10.7554/eLife.77335)
Supplement: Figure 2—source data 1. [file elife-77335-fig2-data1.zip › Figure_2_source_data_1/Figure_2I_J/homerResults/motif48.similar.html]

motif48

## Information for motif48

A
G
T
C
T
G
C
A
A
G
T
C
C
G
T
A
A
G
T
C
A
C
G
T
A
G
T
C
C
G
T
A
  
Reverse Opposite:  

A
C
G
T
A
C
T
G
G
T
C
A
A
C
T
G
A
C
G
T
A
C
T
G
A
C
G
T
A
C
T
G
  

|  |  |
| --- | --- |
| p-value: | 1e-19 |
| log p-value: | -4.433e+01 |
| Information Content per bp: | 1.929 |
| Number of Target Sequences with motif | 28.0 |
| Percentage of Target Sequences with motif | 3.86% |
| Number of Background Sequences with motif | 1.6 |
| Percentage of Background Sequences with motif | 0.58% |
| Average Position of motif in Targets | 87.0 +/- 40.5bp |
| Average Position of motif in Background | 119.0 +/- 19.9bp |
| Strand Bias (log2 ratio + to - strand density) | -0.1 |
| Multiplicity (# of sites on avg that occur together) | 1.07 |
| Motif File: | file (matrix) reverse opposite |

### Similar de novo motifs found

|  |  |  |  |  |  |  |  |
| --- | --- | --- | --- | --- | --- | --- | --- |
| Rank | Match Score | Redundant Motif | P-value | log P-value | % of Targets | % of Background | Motif file |
| 1 | 0.631 | A G T C A C T G C G T A A T C G G C A T C T G A C A G T A T G C | 1e-15 | -35.069954 | 6.20% | 1.72% | motif file (matrix) |
